# Characterization of a population of neural progenitor cells in the infant hippocampus

**DOI:** 10.1111/nan.12065

**Published:** 2014-07-01

**Authors:** S M L Paine, A R Willsher, S L Nicholson, N J Sebire, T S Jacques

**Affiliations:** * Neural Development Unit, Birth Defects Research Centre, UCL Institute of Child HealthLondon, UK; † Department of Histopathology, Great Ormond Street Hospital for Children NHS Foundation TrustLondon, UK

**Keywords:** dentate gyrus, doublecortin, hippocampus, neural progenitor, polymorphic layer

## Abstract

**Aims:**

Abnormalities of the hippocampus are associated with a range of diseases in children, including epilepsy and sudden death. A population of rod cells in part of the hippocampus, the polymorphic layer of the dentate gyrus, has long been recognized in infants. Previous work suggested that these cells were microglia and that their presence was associated with chronic illness and sudden infant death syndrome. Prompted by the observations that a sensitive immunohistochemical marker of microglia used in diagnostic practice does not typically stain these cells and that the hippocampus is a site of postnatal neurogenesis, we hypothesized that this transient population of cells were not microglia but neural progenitors.

**Methods:**

Using archived *post mortem* tissue, we applied a broad panel of antibodies to establish the immunophenotype of these cells in 40 infants dying suddenly of causes that were either explained or remained unexplained, following *post mortem* investigation.

**Results:**

The rod cells were consistently negative for the microglial markers CD45, CD68 and HLA-DR. The cells were positive, in varying proportions, for the neural progenitor marker, doublecortin, the neural stem cell marker, nestin and the neural marker, TUJ1.

**Conclusions:**

These data support our hypothesis that the rod cells of the polymorphic layer of the dentate gyrus in the infant hippocampus are not microglia but a population of neural progenitors. These findings advance our understanding of postnatal neurogenesis in the human hippocampus in health and disease and are of diagnostic importance, allowing reactive microglia to be distinguished from the normal population of neural progenitors.

## Introduction

Abnormalities of the hippocampus have been linked to a range of pathological states in children, including epilepsy 1 and sudden death 2,3. Clusters of rod-shaped cells are frequently seen at *post mortem* in infants in part of the hippocampal complex, the polymorphic layer (PML) of the dentate gyrus (DG). The PML is the inner layer of the three that make up the DG and lies between the granule cells of the DG and the pyramidal cells of CA4 4. On the basis of morphology (including ultrastructure), lectin histochemistry and immunohistochemistry for HAM56, it has been reported that these rod cells in the PML of infants were microglia 5. These cells were rarely seen beyond 9 months of corrected age and were more frequent in infants dying of a range of chronic hypoxic or hypotensive states and in sudden infant death syndrome (SIDS) 5 than in infants dying of acute causes.

The presence of these characteristic cells in the PML of infants dying suddenly has since been interpreted as evidence of hypoxic/ischaemic injury 6,7. Furthermore, the finding in the initial report that rod cells were not seen in the PML of infants dying less than 4 days after brain injury 5 has been cited as evidence that this is the period required for a microglial response to develop 8.

In the course of our diagnostic practice, we made two observations: that the rod cells of the PML were not identified by antibodies directed against CD68, a sensitive marker of microglia routinely used in neuropathological practice, and, consistent with the original report, that the rod cells were only present in infants 5. A reduction in cellularity of the PML between birth and the age of 9 months has also been reported by an independent group 4. Given that the hippocampus is a site of postnatal neurogenesis 9, we hypothesized that this transient population of cells were not microglia but neural progenitors. The aim of this study was to test this hypothesis using a broad panel of immunohistochemical markers in infants dying suddenly of causes that were either explained or remained unexplained following *post mortem* investigations. Clarifying the nature of these cells is important for our understanding of the biology of the infant hippocampus and essential for the practice of diagnostic neuropathology.

## Materials and methods

This study was conducted with the approval of the local research ethics committee (05/Q0508/96). Cases were selected from the archive of Great Ormond Street Hospital if they met the following conditions: after a full *post mortem* examination, including ancillary investigations, the cause of death was either explained or unexplained sudden unexpected death in infancy (SUDI); formalin-fixed, paraffin-embedded hippocampal tissue was available; and, prominent rod cells were present in the PML of the hippocampus. In total 40 cases were included, 20 explained SUDI [eSUDI, aged 0–314 corrected days (postnatal age minus the difference between term and gestational age at birth if preterm), mean age 55 days, 12 males] and 20 unexplained SUDI (uSUDI, aged 18–338 corrected days, mean 95 days, 11 males). The causes of death in the eSUDI group are shown in Table 1. Based on our observations during clinical practice, we predicted that 20 cases in each group would be adequate to characterize the immunophenotype of the rod cells.

**Table 1:** Causes of death in the eSUDI group

Infection, including CNS infection	5
Congenital, non-CNS abnormalities	4
Chronic respiratory disease	3
Hypoxic/ischaemic encephalopathy	3
Non-traumatic intracranial haemorrhage	2
Multi-organ failure	2
Complication of cardiac surgery	1
Total	20

CNS, central nervous system.

An initial cohort of five cases (three eSUDI and two uSUDI cases) was selected at random and from these, 5-μm-thick sections of hippocampus were cut and immunostained, using an automated stainer (Bond-Max Leica, Wetzlar, Germany) as per the manufacturer's instructions, with a panel of 16 antibodies: CD31 (pre-diluted, Leica PA0250), CD34 (pre-diluted, Leica PA0212), CD45 (1:500, Dako M0701, Glostrup, Denmark), CD68 (pre-diluted, Leica PA0273), CD133 (1:25, MACS 130-098-826, Bergisch Gladbach, Germany), doublecortin (DCX, 1:1500, Abcam ab18723, Cambridge, UK), epithelial membrane antigen (EMA, pre-diluted, Leica PA0035), glial fibrillary acidic protein (GFAP, 1:20 000, Dako 20334), human leucocyte antigen-DR (HLA-DR, 1:3000, Dako M0775), Ki67 (pre-diluted, Leica PA0118), microtubule-associated protein 2 (MAP2, Sigma-Aldrich M4403, St. Louis, MO, USA), nestin (1:2000, Millipore AB5922, Darmstadt, Germany), octamer-binding transcription factor 3/4 (Oct3/4, pre-diluted, Leica PA0934), sex determining region Y-box 2 (SOX2, 1:500, Millipore AB5603), neuron-specific class III beta-tubulin (TUJ1, 1:2500, Covance MMS-435P, Greenfield, IN, USA) and vimentin (pre-diluted, Leica SRL33). Appropriate positive controls for each antibody were stained in parallel (Supplementary Figure S1). Negative controls, lacking primary antibody, showed no non-specific staining. Sections of hippocampus from the remaining 35 cases were all immunostained with CD68, CD133, DCX, nestin and TUJ1. A section from each case was stained with haematoxylin and eosin (H&E).

Staining was assessed blind to the cause of death. In every case, one section per stain was assessed either qualitatively (nestin and TUJ1) or quantitatively (DCX). As the development of the two limbs of the DG progresses at different rates 4 and the rod cells are not uniformly distributed, the immunoreactivity of rod cells in the PML of both the superior and inferior limbs of the DG was assessed. Images were taken at × 20 objective, using a Digital Microimaging Device (DMD108, Leica), of the two areas of the PML, where the rod cells were qualitatively assessed to be densest, in both the superior and inferior limbs of the DG for each stain (that is, four fields of view per section). Immunoreactivity for nestin and TUJ1 was assessed qualitatively: the proportion of positive cells was estimated by a neuropathologist, blind to the cause of death, in each of the four fields of view per case. Staining for DCX was assessed quantitatively: the total number of rod cells and the number that were immunoreactive for DCX were counted manually using ImageJ 10 in each of the four fields per case. Statistical analyses were performed using SPSS.

## Results

### The rod cells of the PML of the DG are consistently negative for three sensitive immunohistochemical markers of microglia

**Figure 1:**
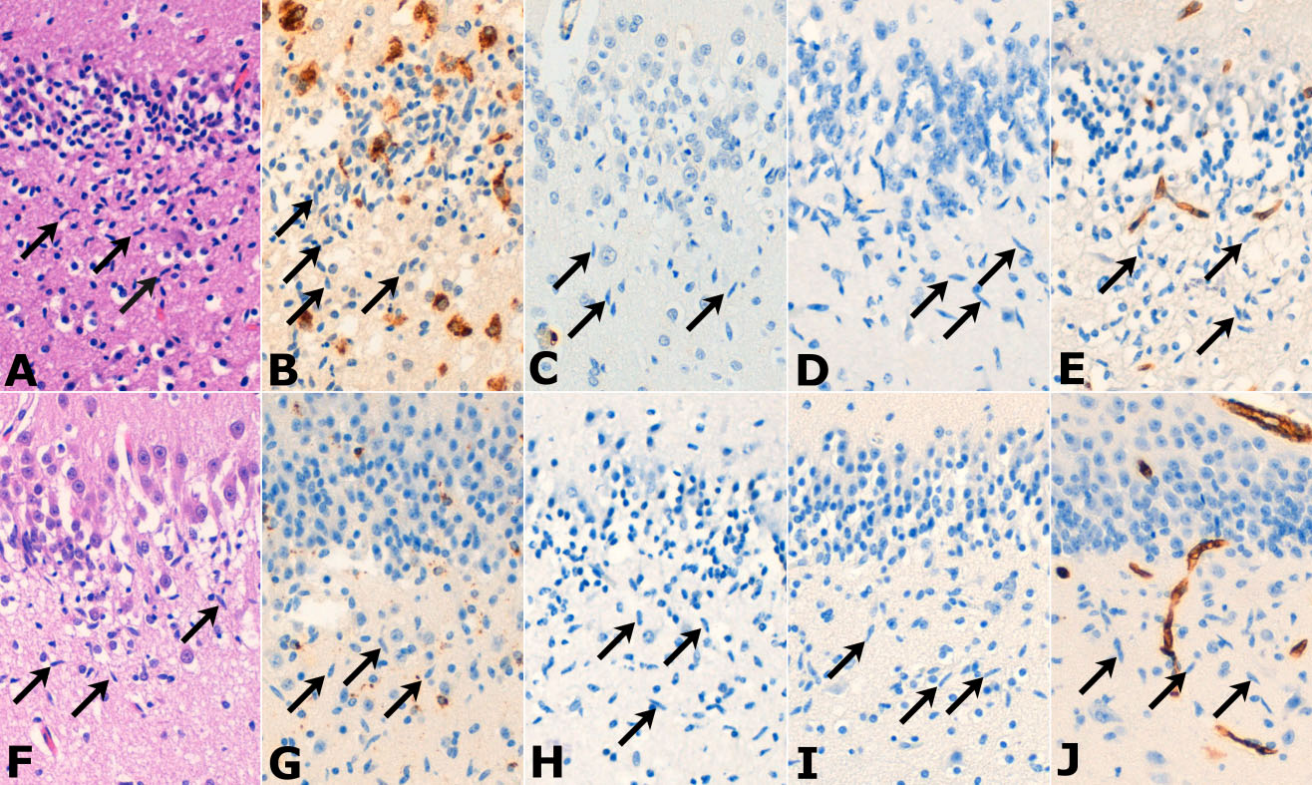
Sections from cases of eSUDI (A–E) and uSUDI (F–J). CD68-positive microglia were present in some cases (B and G), but the rod cells (arrows) of the polymorphic layer of the dentate gyrus were negative for CD68 and the other microglial markers CD45 (C and H) and HLA-DR (D and I). A CD45-positive circulating lymphocyte is seen within a vessel (C). The endothelial cells, but not the rod cells, expressed CD34 (E and J). All images × 20 objective.

The rod cells in the initial cohort of five cases were uniformly negative for the microglial markers CD68, CD45 and HLA-DR (Figure 1). In the larger cohort of 40 cases, all had rod cells present but only six cases of eSUDI and one of uSUDI had significant numbers of infiltrating CD68-positive microglia. However, even in cases where there was a florid CD68-positive microglial response (e.g. established hypoxic/ischaemic injury), a consistent population of rod cells was present that were negative for CD68. CD68 did not stain the rod cells of the PML in any of the remaining cases.

### Irrespective of the cause of death, many of the rod cells of the PML of the DG are positive for immunohistochemical markers of neural progenitor cells

**Figure 2:**
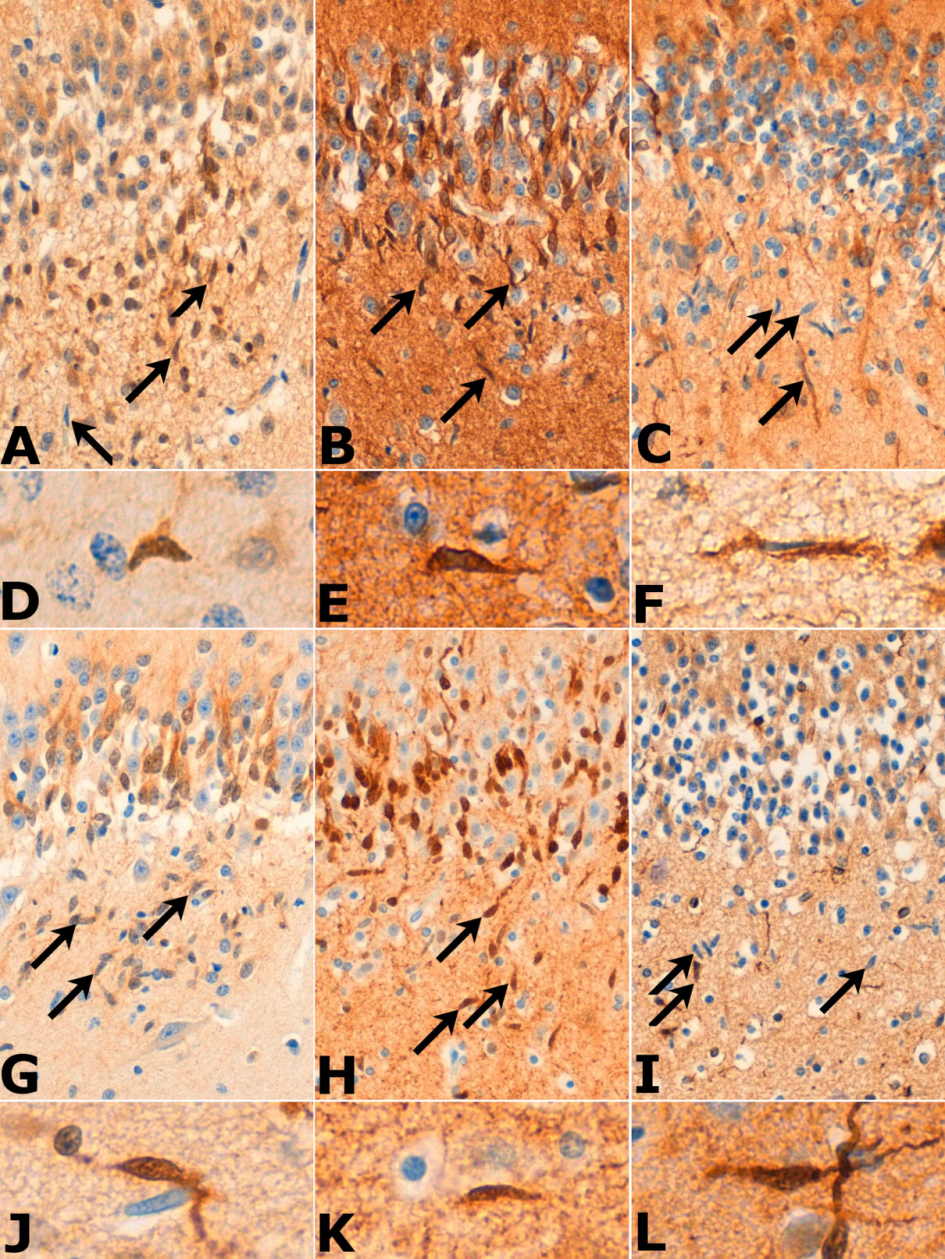
Sections from cases of eSUDI (A–F) and uSUDI (G–L). The majority of the rod cells (arrows) were positive for TUJ1 (B, E, H and K). Between 10% and 30% of the cells expressed DCX (A, D, G and J) and nestin (C, F, I and L). Images A–C, G–I × 20 objective; D–F, J–L × 63 objective.

In all cases, rod cells were immunoreactive for doublecortin, nestin and TUJ1 (Figure 2). The proportions of cells that were stained by nestin and TUJ1 was assessed qualitatively and found to be between 10% and 30% and 60% and 80% respectively. Quantitative assessment of DCX expression showed that in the superior and inferior limbs 13.2% [95% confidence interval (CI) 10.7–15.7%] and 12.5% (95% CI 9.8–15.2%) of rod cells were positive respectively. Occasional rod cells were positive for the proliferation marker Ki67 (Supplementary Figure S2). The rod cells were uniformly negative for the stem cell markers CD133 and Oct3/4 (Supplementary Figure S2). In two of five cases stained, cytoplasmic immunoreactivity to SOX2 was noted in rare rod cells but nuclear staining was not present (Supplementary Figure S2).

Although, some endothelial cells were immunoreactive for nestin, as has previously been reported 11, neither DCX, nor TUJ1 stained the endothelial cells and the rod cells were uniformly negative for the endothelial markers CD31 and CD34 (Supplementary Figure S2). The rod cells were negative for the intermediate filament vimentin, the microtubule-binding protein MAP2, the ependymal marker EMA and glial marker, GFAP (Supplementary Figure S2). Expression of CD133, EMA and GFAP was noted in ependymal cells only, as expected (data not shown).

### Rod cells of the PML are present in infants dying from eSUDI and uSUDI beyond 9 months of corrected age

As cases were selected on the basis that they contained prominent clusters of rod cells in the PML, statistical analysis of the relationship between the number of rod cells and age or cause of death was not possible. However, we did observe that prominent clusters of rod cells were present beyond the age of 9 months, in four cases with corrected ages of 299, 314, 334 and 338 days.

## Discussion

We have described the identity of a population of neural progenitors in the infant hippocampus that have been previously regarded as microglia. Several lines of evidence support this conclusion. First, using a range of sensitive markers of microglia, we have not found evidence of a significant population of microglia in the PML of infants dying of both explained and unexplained SUDI. This is in contrast to the only other study that addressed the identity of these cells 5. The disparity between our findings and those previously reported are likely to be due to the specificity of the different techniques employed. We used three sensitive and relatively specific immunohistochemical markers of microglia in a cohort of five cases and stained the remaining 35 cases with one of these, CD68, which is the marker most commonly used in diagnostic neuropathology. In no case was a significant population of rod cells stained by these markers. In a previous study, 18 cases were stained with lectin histochemistry and the immunohistochemical marker HAM56 5. Histochemistry using RCA-1 and the avidin–biotin peroxidase system has been shown to detect microglia 12 but it also binds endothelial cells 5,12,13. Likewise, the HAM56 antibody reacts with microglia but also macrophages 5 and endothelial cells 14,15. Neither of these techniques has been reported to detect neural progenitor cells. Of note, the ultrastructural analysis conducted in the original report did not demonstrate lysosomes 5, structures which are typically present in microglia 16.

Second, using a range of immunohistochemical progenitor cell markers, we have shown that the rod cells of the PML of the DG in infants have the phenotype of neural progenitor cells. The markers we used are differentiation dependent, identifying pluripotent cells (Oct3/4 17, CD133 18 and SOX2 19), neuroepithelial precursor cells (nestin 20), immature neurones (TUJ1 21) and migrating and differentiating neurones (MAP2 and DCX 22). Some of the earliest progenitors are also immunoreactive for GFAP 23. The majority of the rod cells in the PML are immunoreactive for TUJ1, with a minority staining for the earlier markers nestin and DCX 24, suggesting that they are a population of committed neural progenitor cells with modest variability in differentiation. The presence of occasional Ki67-positive cells, despite the tissue being *post mortem*, confirms that the cells are proliferating. No method capable of detecting neural progenitor cells was used in the previous report 5. Postnatal neurogenesis in the DG is well described, in both experimental animals 25–27 and humans 28 and is implicated in the formation of new memories 29. The previous reports 4,5 and our observations support the hypothesis that the population of cells we have characterized is transient, declining in number towards the end of the first year of life. How these developmental progenitors relate to those seen later in life is an interesting question.

The present data challenge two conclusions which have been drawn on the basis of the previous report of the distribution and lineage of the rod cells of the PML 5. First, the observation that these cells were more numerous in cases of SIDS and in infants dying of a range of chronic hypoxic or hypotensive states 5, has been interpreted as evidence of hypoxic/ischaemic brain injury in SIDS 6,7. We have shown that a population of rod cells in the PML is a normal finding, being present in infants dying of both eSUDI and uSUDI, and that these cells are not microglia but neural progenitor cells. Therefore, for diagnostic purposes, simply commenting on the presence of these cells as evidence of a pathological process, without some form of quantification, is inadequate. If there are increased numbers of these cells in SIDS or hypoxic/ischaemic injury, this may be due to enhanced neurogenesis, either as part of an underlying pathological process or as a reactive phenomenon. While the aims of our study did not include correlating the number of rod cells with the cause of death, given that abnormalities of the neuronal populations of the hippocampus have been associated with sudden death in childhood 2 and epilepsy 1, it is plausible that this population of neural progenitor cells may contribute to specific disease states, including SIDS. Alternatively, given the previous report of increased numbers of rod cells in infants dying of chronic hypoxic or hypotensive conditions 5, they may proliferate in response to various stresses, including hypoxia.

Second, the initial report that rod cells were not observed in infants surviving less than 4 days after brain injury 5 has been interpreted to indicate that a period of survival of 4 days or more is required after an insult for a microglial response to become apparent neuropathologically 8. Determining the timing of a brain injury from neuropathological changes is a frequent and important issue in diagnostic practice. As the predominant immunophenotype of the rod cells in the PML is not microglial, we argue against using the presence of these cells as an indicator of the timing of neuropathological changes. Rather, we suggest that evidence from reports specifically addressing this question should be used 30–32.

In summary, we have shown that a population of cells in the infant hippocampus are not, as previously reported, microglia but neural progenitor cells. This finding is an important step forward in our understanding of postnatal neurogenesis in the hippocampus and has critical implications for the practice of diagnostic neuropathology. The fascinating possibility of a relationship between these cells and particular causes of death needs further work, but may provide a clue to the pathogenesis of diseases, such as SIDS.

## Supporting Information

Figure S1 Positive controls were stained in parallel with the cases and each showed the expected pattern of immunoreactivity. CD31 (brain, **A**), CD34 (brain, **B**), CD45 (tonsil, **C**), CD68 (brain, **D**), CD133 (pancreas, **E**), DCX (brain, **F**), EMA (brain, **G**), GFAP (brain, **H**), HLA-DR (tonsil, **I**), Ki67 (small intestine, **J**), MAP2 (brain, **K**), nestin (brain, **L**), Oct3/4 (germinoma, **M**), SOX2 (focal cortical dysplasia, **N**), TUJ1 (small intestine, **O**), vimentin (brain, **P**). All images × 20 objective.

Figure S2 The rod cells (arrows) were negative for the immunostains CD31 (**A**), CD133 (**B**), EMA (**C**), GFAP (**D**), MAP2 (**F**), Oct3/4 (**G**), SOX2 (**H**) and vimentin (**I**). An occasional rod cell was positive for Ki67 (white arrow, **E**), an epitope that is particularly prone to *post mortem* degradation. All images × 20 objective.
