# Supplementary figures and images for: Characterization of a population of neural progenitor cells in the infant hippocampus
Source: Neuropathol Appl Neurobiol. 2014 Jul 1;40(5):544–50. doi: 10.1111/nan.12065 (PMC4260144; doi:10.1111/nan.12065)

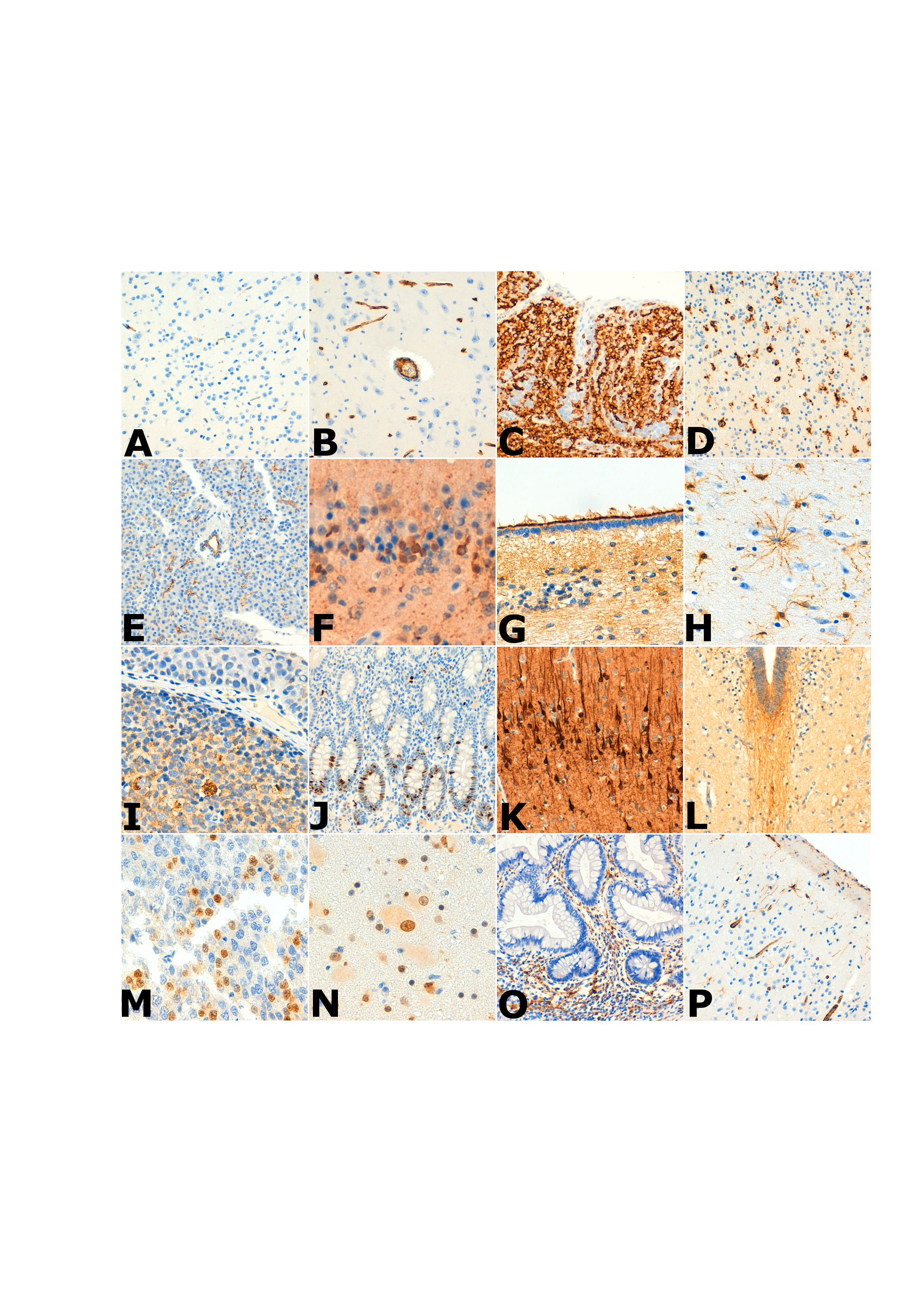

Supplement: Figure S1 — Positive controls were stained in parallel with the cases and each showed the expected pattern of immunoreactivity. CD31 (brain, A), CD34 (brain, B), CD45 (tonsil, C), CD68 (brain, D), CD133 (pancreas, E), DCX (brain, F), EMA (brain, G), GFAP (brain, H), HLA-DR (tonsil, I), Ki67 (small intestine, J), MAP2 (brain, K), nestin (brain, L), Oct3/4 (germinoma, M), SOX2 (focal cortical dysplasia, N), TUJ1 (small intestine, O), vimentin (brain, P). All images × 20 objective. [file nan0040-0544-SD1.tif]

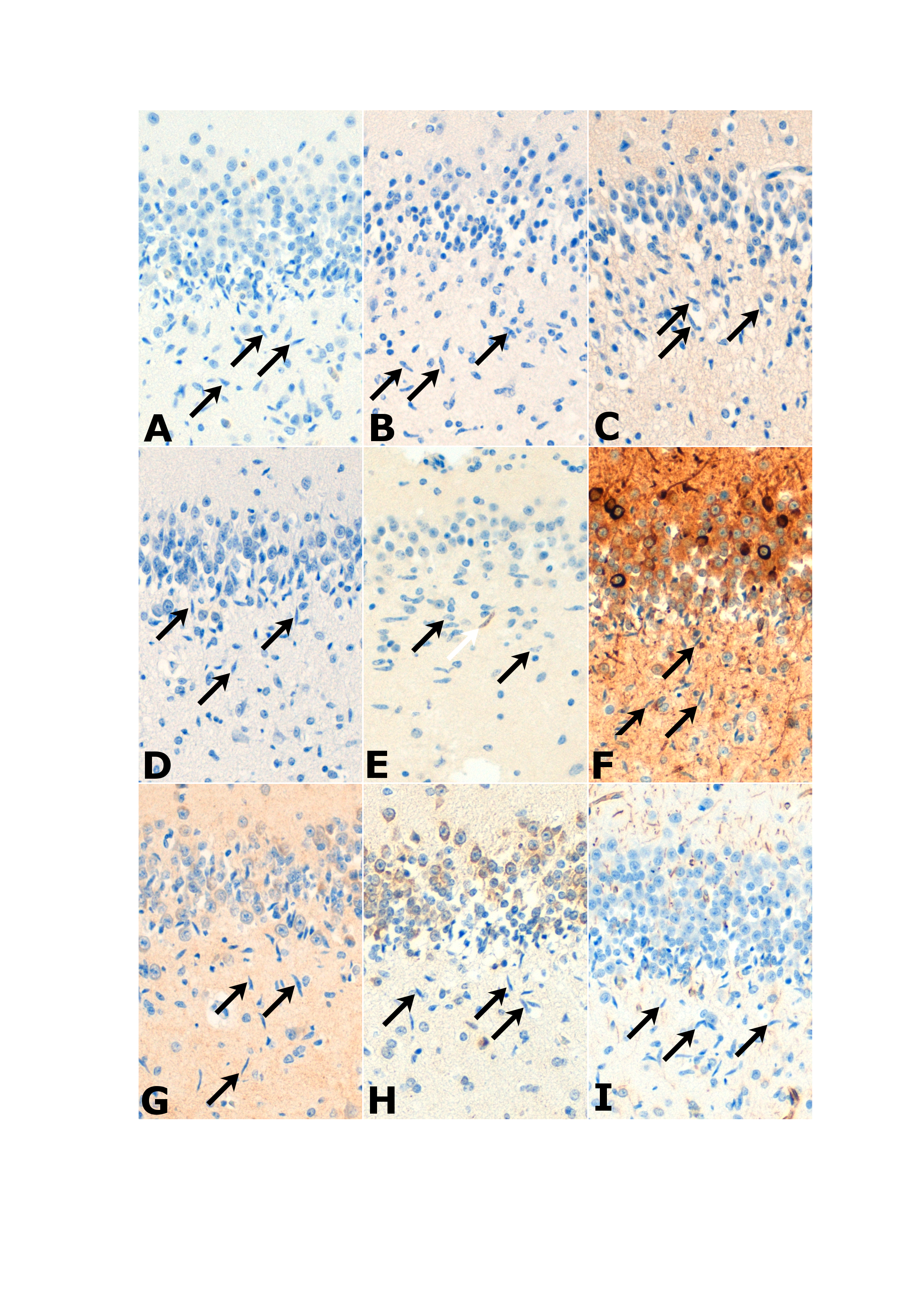

Supplement: Figure S2 — The rod cells (arrows) were negative for the immunostains CD31 (A), CD133 (B), EMA (C), GFAP (D), MAP2 (F), Oct3/4 (G), SOX2 (H) and vimentin (I). An occasional rod cell was positive for Ki67 (white arrow, E), an epitope that is particularly prone to post mortem degradation. All images × 20 objective. [file nan0040-0544-SD2.tif]
